# Oxidative and Nitrosative Stress in the Metastatic Microenvironment

**DOI:** 10.3390/cancers2020274

**Published:** 2010-03-26

**Authors:** Ángel L. Ortega, Salvador Mena, José M. Estrela

**Affiliations:** 1 Department of Physiology, Faculty of Medicine and Odontology, University of Valencia, 15 Av. Blasco Ibañez, 46010 Valencia, Spain; E-Mail: angel.ortega@uv.es; 2 Green Molecular S.L., Pol. Ind. La Coma-Parc Cientific, 46190 Paterna, Valencia, Spain; E-Mail: salvador.mena@uv.es

**Keywords:** metastases, tumor microenvironment, reactive oxygen species, reactive nitrogen species

## Abstract

Metastases that are resistant to conventional therapies are the main cause of most cancer-related deaths in humans. Tumor cell heterogeneity, which associates with genomic and phenotypic instability, represents a major problem for cancer therapy. Additional factors, such as the attack of immune cells or organ-specific microenvironments, also influence metastatic cell behavior and the response to therapy. Interaction of cancer and endothelial cells in capillary beds, involving mechanical contact and transient adhesion, is a critical step in the initiation of metastasis. This interaction initiates a cascade of activation pathways that involves cytokines, growth factors, bioactive lipids and reactive oxygen and nitrogen species (ROS and RNS) produced by either the cancer cell or the endothelium. Vascular endothelium-derived NO and H_2_O_2_ are cytotoxic for the cancer cells, but also help to identify some critical molecular targets that appear essential for survival of invasive metastatic cell subsets. Surviving cancer cells that extravasate and start colonization of an organ or tissue can still be attacked by macrophages and be influenced by specific intraorgan microenvironment conditions. At all steps; from the primary tumor until colonization of a distant organ; metastatic cells undergo a dynamic process of constant adaptations that may lead to the survival of highly resistant malignant cell subsets. In this sequence of molecular events both ROS and RNS play key roles.

## 1. Introduction

Cancer can be viewed as a complex cellular phenotype, which is associated with unlimited replicative potential, independence from growth signals with parallel resistance to growth-inhibitory signaling, evasion of cell death activation, sustained angiogenesis, as well as the ability of tissue invasion and metastasis [1]. Malignant tumors are invasive, and may metastasize to distant sites through the circulatory system. Consequently, metastatic spread, not the primary tumor burden, is the main cause of cancer-related deaths [2]. In fact control and treatment of metastases still represents a major, unsolved, scientific and clinical challenge.

Reactive oxygen species (ROS) are highly reactive molecules that are constantly produced in all aerobic organisms, mostly as a consequence of aerobic respiration. The term covers several types of chemical species, including free radicals such as superoxide (O2**^.−^**) or hydroxyl (OH·), and nonradicals such as hydrogen peroxide (H_2_O_2_). Levels of ROS are reduced by antioxidant defenses, but increased by transition metals such as iron or copper and by exogenous agents such as ionizing radiation or ozone [3]. Similarly, nitrogen-derived free radicals are called reactive nitrogen species (RNS) and their utmost representative precursors are nitric oxide (NO) and peroxynitrite (ONOO^−^) [4]. NO is well known to be a product of the catalytic action of the nitric oxide synthase (NOS) enzyme family on L-arginine [5]. However, recent evidence suggests that it can also be formed by reduction of nitrite, which can arise in the body by ingestion or from bacterial metabolism [6]. Low levels of both ROS and RNS are continuously produced in mammalian cells and play important physiological roles [7]. These include processes as diverse as gene expression [8], cell proliferation and survival [9], pathogen clearance by the immune system, and blood vessel permeability. However, when the amount of ROS/RNS exceeds the capacity of the antioxidant machinery, the resulting oxidative/nitrosative stress may induce irreversible damages in all cellular macromolecules, including genomic DNA (Table 1). Therefore, maintenance of redox homeostasis is critical for cell function and survival, and its alteration is involved in the pathophysiology of many human diseases, such as cardiovascular diseases, diabetes, rheumatoid arthritis, neurological disorders (e.g., Alzheimer and Parkinson disease), or cancer [10,11,12,13,14,15]. 

The complex mechanisms involved in ROS and RNS actions make it difficult to treat the different events independently. This fact is particularly relevant when considering the influence of these reactive species in the pathophysiology and therapy of human cancers. Protein and DNA damage induced by ROS are linked to the failure to repair DNA damage, *i.e.*, in K-ras mutation (12 G-T trasversions). For instance, these types of mutations are an early event in the development of adenocarcinoma of the lung and are present in 30% or more of all cases [16,17]. Besides recessive loss-of-function mutations in tumor suppressor genes such as p53 and p16, or dominant gain-of-function in oncogenes, chromosomal abnormalities, DNA adduct formation, methylation, and acetylation are characteristic of lung cancer and of other human tumors [18,19]. ROS and RNS have an active role in the induction of some of these alterations. In fact, levels of a hydroxyl-mediated DNA adduct, 8-hydroxydeoxyguanosine, are elevated in mice treated with NNK[4-(methylnitrosamino)-1-(3-pyridyl)-1-butanone], a tobacco-specific carcinogen [20]. Moreover, Jun oncogene stimulation by ROS and RNS has also been linked to lung cancer [21,22]. Oxidative modification of lipids induces products that react with DNA. Protein oxidation promotes mutagenesis through DNA polymerase alteration or inhibition of DNA repair enzymes [23,24].

Clinical and epidemiological research has provided strong evidence supporting the role of ROS and RNS in the etiology of cancer, due to different factors such as solar UV exposure [25,26], chemical carcinogens, lifestyle, diet and environment, or chronic inflammation conditions [27,28]; where high levels of free radicals are produced. Mutations in cancer-related genes, or post-translational modifications of proteins by nitration, nitrosation, phosphorylation, acetylation or poly ADP-ribosylation by free radicals or lipid peroxidation (Table 1) by products such as the reactive aldehydes malondialdehyde (MDA) and 4-hydroxy nonenal (HNE), are some key events that may increase cancer risk [29]. Indeed, free radicals can increase DNA mutation rates to levels comparable to those promoted by other well-known carcinogens (*i.e.*, polycyclic aromatic hydrocarbons, or aflatoxins), and thus are considered powerful cancer initiators [30]. *In vivo* experiments with animal models also support a causal role for free radicals in cancer. Indeed knock-out mice for distinct antioxidant enzymes that regulate ROS levels *in vivo* (*i.e.*, superoxide dismutase, SOD; and GSH peroxidase, GPx) not only have higher levels of ROS in their tissues, but also suffer from higher rates of spontaneous tumors [31]. Similarly, mice deficient in Mth1, a key enzyme involved in the repair of DNA oxidative lesions, also show higher rates of spontaneous lung, liver, and stomach tumors [32].

**Table 1:** Molecular damages induced by ROS and RNS.

DNA	
Point mutations	[33]
DNA-DNA and DNA-protein crosslinks	[33]
Sister chromatid exchanges	[33]
Single- or double-strand breaks	[33]
Increased 8-HO-dG levels with G-T transversions	[34]
Other oxidation-derived products such as 5-hydroxy-dC, 5-hydroxy-dU and uridine glycol with C-T transitions	[33]
Proteins	
Amino acid oxidation	
Post-translational modifications	[35]
eNOS mediated Ras activation by S-nitrosylation	[36]
Lipids	
Direct oxidation of polyunsaturated fatty acids present in lipids	[37]
Indirectly by lipid synthesis inhibition, fatty acid desaturation, or lipase activation	[37]
Iron-mediated decomposition of lipid hydroperoxides can yield a plethora of follow-up products such as conjugated dienes, hydrocarbon gases (e.g., ethane, ethene) and carbonyl compounds such as malondialdehyde (MDA), alkenals, alkadienals, and α,β-unsaturated aldehydes (e.g., crotonaldehyde, acrolein). New studies on autoxidation of arachidonic acid revealed that intermediate formation of monocyclic peroxides, bicyclic endoperoxides, and dioxolane-isoprostane peroxides may also occur.	[36,38]

Furthermore, ROS have been shown to promote proliferation of various cancer cell types *in vitro* [39,40], which highlights their cancer promoting potential. Exposure of several cancer cell lines to inflammation- or chemically-induced ROS boosts their migratory and invasive behaviors [40,41,42], hence suggesting a role of free radicals in favoring the invasive phenotype. In fact, a growing body of evidence suggests that many cellular responses to oxidative and nitrosative stress are indeed regulated at the transcriptional level [43]. Nitrosylation or oxidation of critical Cys residues in the DNA-binding domains or at allosteric sites may regulate transcription of target (malignancy-related) genes [43]. On the other hand, it is also known that exposure to free radicals above a certain threshold irreversibly leads to cell damage (Table 1), and eventually to cell death [44,45]. ROS and RNS also appear to be critical for the tumoricidal activity of the immune system [27,46,47]. Furthermore, several cancer chemotherapeutic agents (*i.e.*, cisplatin or arsenic trioxide), as well as radiotherapy, are known to exert their cytotoxic effects through ROS-mediated mechanisms [44]. Therefore, the net result of pro- and anti-cancer ROS and RNS effects may likely determine the rate and extent of *in vivo* tumor progression. In this review, we will focus on the role of ROS and RNS in regulating cancer cell dynamics and survival in the metastatic microenvironment, where interaction with endothelial cells, extravasation, growth, and angiogenesis are critical steps in the process of metastatic invasion.

## 2. Metastases

### 2.1. Biology and the Seed and Soil Hypothesis

Tumor formation and metastasis classically includes DNA damage and mutagenesis, causing transformation of normal cells into preneoplastic cells (initiation), followed by selective clonal expansion (promotion), and a second mutagenic mechanism responsible for the ability of some malignant cells to acquire more aggressive characteristics (progression). Malignant tumors are invasive and may reach distant sites through the circulatory system [48]. The classical simplification of metastasis in local invasion, intravasation, survival in the blood and lymphatic system, extravasation, and colonization, has helped to understand and rationalize the complex set of factors and properties that must be acquired by a cancer cell in order for it to be considered malignant [49]. In practice, although cancer is a genetic disease, mutagenic transformations are not sufficient to acquire metastatic competence, in fact many oncogene-driven mouse models of cancer are not able to automatically establish metastases [50]. 

Although seeding can occur in multiple organs, in many cases metastatic tumors grow only in one or just in a few [51]. Steven Paget’s “seed and soil” hypothesis introduced the concept that a receptive microenvironment is required for the development of metastasis [52]. Previously to Paget hypothesis, it was thought that tumor dissemination was determined by mechanical factors that caused tumor cell emboli in the vasculature [53]. However, now we know that the microenvironment clearly has important effects on tumor and metastasis development. There is evidence that cancer cells are able to remain in a dormancy state (a state of cellular quiescence in the G0 phase of the cell cycle) even for many years [49,54,55,56], or remain in a balanced state of proliferation and apoptosis [49,57]. The microenvironment may suppress the malignancy of potentially metastatic cells, but likewise their reactivation to form a new tumor or metastasis probably occurs through perturbations in the microenvironment [58].

#### 2.1.1. Tumor Microenvironment

The complex and paradoxical role of ROS and RNS in tumorigenesis and metastasis have long been studied [59,60]. Early studies associated inflammation and carcinogenesis. In 1863 Rudolf Virchow already noticed the presence of leukocytes in neoplastic tissues [61]. Since Virchow’s observations, many different studies have supported that tumors can originate at the sites of chronic inflammation or infection [62]. Later studies have shown the relationship between NO and the immune response of macrophages [63], or how deletion or expression of inducible NOS (iNOS) can regulate development or growth of several types of cancer [64,65,66]. Altogether, these observations suggest that ROS and RNS have multiple physiological and pathological effects, depending on the tumor microenvironment, concentration, and spatial and temporal constraints.

#### 2.1.2. Tumorigenesis

Environmental agents such as cigarette smoke, xenobiotics, lifestyle, diet, chronic ultraviolet B exposure, and sustained cellular injuries can generate ROS and RNS production, which can function as chemical effectors in tumorigenesis [25,26,37,67,68,69]. DNA damage (Table 1), leading to activation of oncogenes and/or non-expression of tumor suppressor proteins, is one of the plausible mechanisms by which ROS and RNS can promote carcinogenesis. Mutations in the oncogene *RAS* and tumor suppressor gene *P53* have been observed in many types of human cancers [70,71]. Transition-type mutations at dipyrimidine sites and G:C to T:A transversions, in addition of being induced by the presence of 8-oxoguanine during DNA replication [37], are observed in *RAS* and *P53* genes in human skin cancers of sun-exposed areas and in UV-induced mouse skin cancers [72]. Furthermore, ROS and RNS induce protein and cellular membranes damages (Table 1). Occasionally, oxidation and nitrosation may represent an advantage for tumor survival, proliferation stimulation and cell death inhibition [73]. *In vivo* studies have shown that knock-out mice for distinct antioxidants enzymes (*i.e.,* SOD and GPx) not only having higher levels of ROS in their tissues, but also higher rates of spontaneous tumors [31].

The role of intracellular redox state in regulating growth, cell signaling, and/or gene expression is becoming recognized as an important issue. Indeed, alterations in receptor or cytoplasmic tyrosine kinases, levels of specific growth factors, intracellular processes for conveying membrane signals to the nucleus, or the regulation of DNA replication, have been shown in neoplastic cells [15].

#### 2.1.3. Invasion

ROS- and RNS-induced post-translational modifications of proteins regulate a large variety of cellular functions and signaling events. Accumulating evidence shows that free radicals play an important role in cell invasion. In fact, exposure of several cancer cell lines to inflammation- or chemically-induced ROS boost their migratory and invasive behavior [40,41,42]. Epithelial mesenchymal transition (EMT) is not only a physiological mechanism for development and tissue remodeling, but also a pathological mechanism associated with various diseases including inflammation, fibrosis and cancer [74]. During EMT, cell-cell molecular adhesion is decreased, whereas cell-extracellular matrix adhesion is increased, which favors cell migration and invasion. ROS and NO play a pivotal role in the cell-cell dissociation process, since they can regulate the activity of Src kinase. Src is known to be activated in several cancers, and there is convincing evidence that increased Src activity is associated with a more invasive and aggressive phenotype [75]. A number of studies suggest that Src also plays an important role in the cellular response to ROS, because Src-specific inhibitors and dominant-negative Src mutants strongly attenuate cellular response to ROS [76,77,78,79]. Whether ROS activate or inactivate Src is controversial, since it participates in the regulation of numerous cellular processes such as cell survival, cytoskeleton reorganization, DNA synthesis, and cell division [80,81]. Different oxidation loci seem to plausibly explain Src activity: oxidation of Cys_277_ leads to the formation of a disulfide homodimer that inactivates Src activity [82], whereas mutations in Cys_483_, Cys_487_, Cys_496_, and Cys_498_ abolish inactivation of the kinase by known inhibitors binding to cysteine residues such as the SH-alkylating agents BIMP and NAM or by mercury ions [83,84,85]. In addition, cysteine oxidation is important for regulation of the Src kinase after integrin-mediated cell adhesion [86]. Furthermore, ROS induce cell-cell dissociation by endocytosis of N-cadherin mediated by Src kinase phosphorylation and internalization of p120-catenin, which implies loss of epithelial integrity and transient Rho/Rho kinase pathway activation (as it occurs in the initial phase of EMT). The H_2_O_2_-induced Src activation also induces activation of NF-κB leading to *MnSOD* expression, which reduces oxidative stress. This indicates that oxidative stress-induced cell-cell dissociation might be required for the initial step of EMT, but is not sufficient for stable induction of EMT [87]. 

NOS activity has been associated with tumor growth and tumor grade. Nitrosylation of *RAS* has been related to initiation and maintenance of tumors [36]. Moreover, NO may activate c-Src through cysteine modification [88]. NO causes S-nitrosylation of c-Src at Cys_498_ to stimulate its kinase activity resulting in tumor promotion and invasion [89]. For instance, c-Src activation by β-estradiol, observed in breast cancer MCF7 cells, depended on production of NO. Furthermore, this NO-mediated Src activation was critical for reducing the levels of E-cadherin and disrupting cell-cell contacts after β-estradiol stimulation [89]. Loss of E-cadherin activates diverse pathways inducing cell invasion [90]. 

## 3. Interaction between Metastatic and Endothelial Cells

### 3.1. Adhesion, Death, and Survival

Tumor metastasis is attributed not only to the abnormalities of cancer cells, but also to changes induced by the interaction of cancer cells with the surrounding cells/tissues. Indeed, metastases formation involves interactions between tumor cells and a changing microenvironment, which influences cell proliferation, migration, invasion, colonization, and survival [91,92]. 

**Figure 1:**
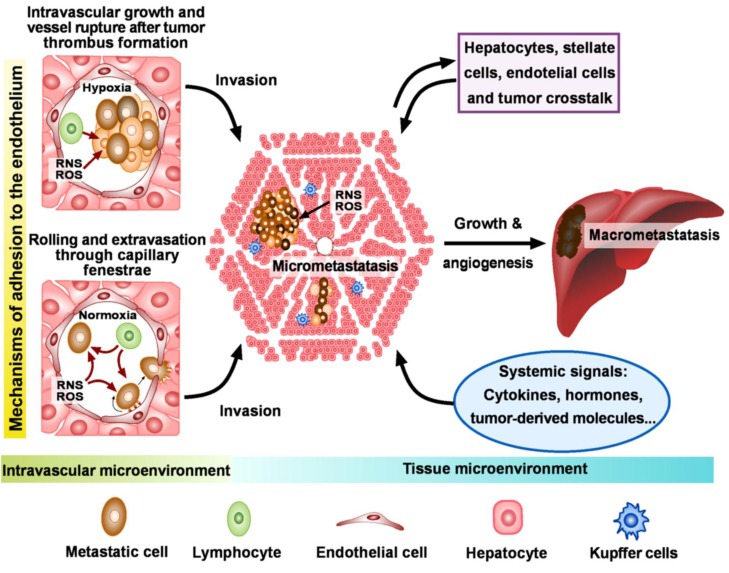
Invasion of the liver by metastatic melanoma cells. Circulating cancer cells attached to the endothelium of pre-capillary arterioles or capillaries may follow two mechanisms of extravasation: (a) intravascular proliferation, formation of a tumor thrombus, and eventual vessel rupture and microinflammation; (b) rolling and migration through vessel fenestrae. Invading cancer cells will then form micrometastases within the normal lobular hepatic architecture, in a mechanism regulated by cross-talk with the stroma. Angiogenesis activation will facilitate metastases grow and spread. High ROS and RNS levels released by the vascular endothelium cause tumor cytoxicity before extravasation, however once organ colonization starts, lower ROS and RNS levels may favor metastatic growth.

Cancer cells that survive the circulatory system and reach different organs/tissues interact with the vascular endothelium before extravasation to begin secondary colonization (Figure 1). Two mechanisms have been proposed to explain this process [93]. Using fluorescence-tagged tumor cells and video-capturing image techniques, Weiss *et al.* found that many tumor cells injected intraperitoneally into mice were arrested in capillaries [94]. Tumor cells often aggregate with platelets, and due to their mass they were observed physically trapped in the capillaries (Figure 1). Arrested tumor cells could remain apparently inactive (senescent?), start growing and extravasate by secreting proteolytic enzymes and rupturing the blood vessel [95], or die due to deformation and surface-membrane rupture [94]. NO was found to inhibit the aggregation of platelets *via* a cGMP-dependent mechanism [96]. In fact, although the ability of metastatic cells to form aggregates with platelets correlates with their metastatic potential, it is inversely proportional to NO generation [97].

Alternatively the mechanism of metastatic cell extravasation may mimic the infiltration of leukocytes to the inflammatory site, in a process requiring adhesion of tumor and endothelial cells (Figure 1). Because many cancer cells express adhesion molecules, which are also expressed on the migrating leukocytes [98], it is generally accepted that metastatic cells use a similar strategy for adhesion to the endothelial cells during metastatic pre-invasion. In this sense, chemokines are important constituents of the tumor microenvironment at metastatic sites, dictating directionality of chemokine receptor-expressing tumor cells, facilitating their adhesion and extravasation, and eventually contributing to organ selectivity [99]. Therefore, although mechanical trapping at the capillaries is indeed observed, the biochemical organ microenvironment presumably plays a critical role in attracting circulating tumor cells to specific microcirculatory areas [100].

Adhesion of metastatic cells to the vascular endothelium is a mechanism mediated by several factors, such as cell adhesion molecules (integrins, selectins, cadherins), immunoglobulins, and cytokines [101,102,103].

Initial contact between metastatic cells and the endothelium (“docking’’) is weak and transient, and likely mediated by carbohydrate–carbohydrate recognition [104]. For instance, interaction between murine B16 melanoma (B16M; widely used as an experimental model to study the metastatic process) and the hepatic sinusoidal endothelium (HSE) involves mannose receptor–mediated melanoma cell attachment to the HSE, which subsequently causes proinflammatory cytokine release (TNF-α, IL-1β, and IL-18), and the VCAM-1–dependent adherence (reinforcing or ‘‘locking’’ the initial intercellular binding) [105]. Murine B16M variant cell lines with low (B16-F1) or high (B16-F10) survival and growth potential *in vivo* are frequently used in experimental metastases research. The B16M is a rapidly growing anaplasic tumor that produces melanin and grows indefinitely *in vitro* under appropriate conditions [106]. After inoculation of this tumor into syngenic mice, it can easily colonize different organs. Some metastatic B16M cells expressed high levels of the integrin VLA-4, the ligand for VCAM-1 on activated endothelial cells [107,108]. Endothelial cells, upon exposure to cytokines released during interaction with metastatic cells, undergo profound alterations of their function that involve gene expression changes, *de novo* protein synthesis, and production of ROS and RNS [104]. In the B16M model, HSE releases large amounts of ROS in response to endotoxins and IL-1. Such pro-inflammatory mediators promote cancer cell adhesion, invasion and proliferation. In fact, *VCAM-1* gene expression in HSE is coupled to an oxidative stress-dependent mechanism [109,110]. Rolling and early adhesion of B16M cells to the HSE, IL-1-dependent endothelial release of H_2_O_2 _through IL-18, and late adhesion of surviving melanoma cells are sequential steps during B16M cell attachment to the HSE that occur in a short period (3–6 h), and enhance melanoma cell adhesion. These mechanisms would likely compensate for ROS-induced direct cytotoxic effects on adherent, vulnerable melanoma cells, leading to the metastatic progression of H_2_O_2_-resistant melanoma cells [111,112]. Low micromolar levels of ROS, which act as intra- and/or intercellular messengers promoting growth and angiogenesis [113,114,115], would then benefit these H_2_O_2_-resistant metastatic cells during their secondary colonization [116]. In agreement with these facts, ROS scavengers have been proposed to be therapeutically effective in suppressing metastasis once secondary growth starts after extravasation [117].

Nevertheless, at this point, it is important to remark that immune cells are also present in the metastatic microenvironment. Both innate and adaptive immunity participates in anti-tumor effects, including the activity of natural killer (NK) cells, natural killer T cells, macrophages, neutrophils and eosinophils, complement, various cytokines, specific antibodies, and specific T cytotoxic cells. Upon activation, neutrophils and macrophages are able to kill tumor cells, but they can also release ROS, angiogenic and immunosuppressive substances [27]. Indeed, inflammatory cells and immunomodulatory mediators present in the tumor microenvironment polarize the host immune response toward specific phenotypes impacting tumor progression [118].

Moreover, exposure of metastatic cells to ROS levels, which may be somehow in the middle between high (necrotic) and low (signaling) concentrations, could also induce cellular senescence and apoptosis and therefore function as anti-tumorigenic species [15]. This can certainly occur in the metastatic microenvironment where gradients of ROS are expected.

On the other hand, NO signaling has been also involved in regulating tumor proliferation, apoptosis, adhesion, migration, invasion, and angiogenesis [119,120]. Indeed, in many different types of cancer, expression of NO synthases, mainly the iNOS isoform, has been positively correlated with tumor invasion and angiogenesis [121,122,123]. However, NO may regulate cancer cell adhesion to the vascular endothelium either positively or negatively (e.g., [124]), a fact likely due to the variability in the NO concentration in tissues, either within cells or at the extracellular compartments. NO has been shown to inhibit adhesion to extracellular matrix components of many cells, including, eosinophils [125] and neutrophils [126]; whereas N-nitro-L-arginine (a general NOS inhibitor) or aminoguanidine (a specific iNOS inhibitor) increased neutrophil adhesion to endothelium [127]. In addition, NO was also shown to inhibit expression of cell adhesion molecules (e.g., ICAM-1) [128]. However, NO may have pro-adhesive effects as shown, for example, in rat brain microvascular endothelial cells, where expression of *ICAM-1* was upregulated synergistically by VEGF and NO [129]. This process was mediated through a phosphatidylinositol-3-OH-kinase (PI3K)/AKT pathway: VEGF caused phosphorylation of AKT by PI3K, leading to production of NO [129].

In addition, a natural defense mechanism against cancer metastasis has been detected, whereby the arrest of tumor cells in the liver induces endogenous NO release, leading to sinusoidal tumor cell killing and reduced hepatic metastasis formation [130]. Based on this evidence, generation of NO concentrations in the high micromolar range, by NO donor drugs or gene therapy with iNOS, has been shown to inhibit tumor growth [120,131]. Nevertheless, the role of iNOS during tumor development is highly complex and is still not completely understood. Both promoting and deterring actions have been described, presumably depending upon the local concentration of iNOS within the tumor microenvironment. In particular, pivotal effects such as malignant transformation, angiogenesis, and metastasis are modulated by iNOS. On the other hand, NO derived from macrophages has a potentially cytotoxic/cytostatic effect on tumor cells [132].

Within the tumor microenvironment, NO can be produced by several cells (tumor cells, macrophages, endothelial or stromal cells). Isolated HSE cells treated *in vitro* with TNF-α and IFN-γ, a manoeuvre that mimics the proinflammatory scenario of the metastatic microenvironment, release NO and H_2_O_2_ in the culture medium [133]. Co-culture of these endothelial cells with B16M cells showed that, during the adhesion process, most of the NO and H_2_O_2_ were generated by the HSE [133]. Moreover, endothelial NO and H_2_O_2_ caused a high percentage of tumor cell death in a concentration-dependent manner [133]. Interestingly, inhibition of NO production using HSE cells isolated from eNOS-deficient (eNOS^-/-^) mice (which abolished eNOS-dependent NO production) or L-NAME (an inhibitor of all NOS activities) showed that H_2_O_2_ released by the HSE did not induce tumor cytotoxicity [133]. However, NO was particularly tumoricidal in the presence of H_2_O_2_ because addition of exogenous catalase, which eliminates H_2_O_2_ released to the extracellular medium, decreased significantly tumor cytotoxicity [133]. These findings are in agreement with later reports showing inhibition of B16M lung metastasis by local release of eNOS-derived NO [134]. When we explored the chemical mechanisms by which NO and H_2_O_2_ are cytotoxic, we found that a major part of the effect requires the presence of trace metals capable of generating highly oxidant radicals, likely •OH and –OONO [133,135].

Therefore, it appears evident that the net production of NO and H_2_O_2_ released during the interaction between metastatic cells and the endothelium is critical to explain their pro- and anti-metastatic effects.

### 3.2. Identification of Key Targets

The liver is a major metastasis-susceptible site and represents an excellent organ for metastatic microenvironment-related studies. In the liver, as well as in other organs/tissues, some cancer cells resist and possibly deactivate anti-tumoral defense mechanisms, likely ignoring growth-inhibitory factors while responding to proliferation-stimulating factors released from tumor-activated hepatocytes, stellate and sinusoidal cells [104]. This leads initially to avascular micrometastasis generation in periportal areas of hepatic lobules. Hepatocytes and myofibroblasts derived from portal tracts and activated hepatic stellate cells are next recruited into some of these avascular micrometastases. These create a microenvironment that supports metastases development through the specific release of both proangiogenic factors and cancer cell invasion- and proliferation-stimulating factors [136].

As previously stated, ROS and RNS contribute to the elimination of circulating and capillary-arrested cancer cells [105,133]. Therefore, it appears plausible that antioxidant defenses may contribute to metastatic cell survival. In the B16M model, some preliminary observations support this possibility: B16M cells pretreated *in vitro* with the lipophilic antioxidant tocopherol (vitamin E) increased their survival in the hepatic sinusoids [137]; an increase in B16M cell glutathione (γ-glutamyl-cysteine-glycine; GSH, the most prevalent non protein thiol in cells) content upon hydroxyurea treatment also transiently increased metastasis [138]; capillary survival decreased in GSH-depleted B16M cells [139]; whereas B16M cells with high GSH content showed higher metastatic activity in the liver than those with lower GSH content [140]. In parallel experiments it was observed that a high % of B16M cells with high GSH content survived combined HSE-derived nitrosative and oxidative attack *in vitro*, and thus, these surviving cancer cells may likely represent the main task force in the metastatic invasion [133]. GSH appears directly involved in regulating metastatic cell survival *in vivo*, because GSH ester (which readily enters the cell and delivers free GSH [141]) pretreatment of B16M-F1 (a variant with low metastatic activity) increased their GSH by four-fold (reaching GSH values similar to those found in B16M-F10 cells) and the % of arrested intact cells in the liver sinusoids after intrasplenic inoculation [142]. Interestingly, invasive B16M-F10 (iB16M) cells, isolated after *in vitro* interaction with the HSE, showed a NO/H_2_O_2_-dependent GSH depletion and a decrease in γ-glutamylcysteine synthetase (γ-GCS) activity [142]. However, overexpression of γ-GCS heavy and light subunits led to a rapid recovery of cytosolic GSH [142]. Thus, since GSH content and metastatic growth appear to be directly related, maintenance of high intracellular levels of GSH may be critical for the extravascular growth of those metastatic cells that survive after interaction with the endothelium.

NO/H_2_O_2_ damage also induces a transient impairment of the mitochondrial system for GSH uptake in invasive cells, and in addition, a decreased activity of respiratory complexes II, III, and IV, less O_2_ consumption and ATP levels, higher NO and H_2_O_2_ production, and lower mitochondrial membrane potential [142]. This is important, because mitochondria do not synthesize GSH [143] and mitochondrial GSH (mtGSH) depletion facilitates permeability transition pore complex (PTPC) opening, and the release of apoptosis-inducing molecular signals [144]. In fact, by using *in vitro* diethylmaleate or monochlorobimane (as thiol-depleting agents), we observed that B16M-F10 cells with low mtGSH levels were highly susceptible to TNF-α-induced oxidative stress and death [142]. Therefore, specific depletion of the mtGSH pool may be a critical target to challenge survival of invasive cancer cells. 

Furthermore, it was observed that different B16M cell line subsets, containing similar GSH levels, showed different rates of survival after *in vivo* interaction with the HSE [142]. Therefore, although GSH content status appears an important parameter for metastasis progression, other factors must also contribute to the survival of some cell subsets with high metastatic potential. Interestingly, the development of resistance to GSH depletion-induced cytotoxicity in CC531 colon carcinoma cells was shown to be associated with increased expression of the anti-apoptotic protein Bcl-2 [145]. Moreover, different reports indicated that increased Bcl-2 levels (in melanoma and other cancer cell types) associated with a concomitant increase in the intracellular GSH content (see e.g., [105]). Thus, a possible link between Bcl-2 and GSH in blocking metastatic cell death appeared plausible. 

The Bcl-2 family members are characterized by the presence of one or several Bcl-2 homology domains and include pro-death and anti-death proteins [146]. Bcl-2 itself is an anti-death protein, and its over-expression has been linked to cancer development, metastatic growth, and chemotherapy resistance [147,148,149,150,151]. On the other hand, expression of pro-death genes, e.g., *BAX* or *BAK*, is often reduced in cancer cells [152].

Regulation of Bcl-2 protein levels may include transcriptional and post-transcriptional control, protein translocation, and protein-protein interactions [153]. However, in cancer cells, whereas some reports show evidence for post-transcriptional down-regulation of *BCL-2* (e.g., [154,155]), others demonstrate an overproduction of the Bcl-2 protein on the basis on increased *BCL-2* mRNA levels (e.g. [156,157]). A possible explanation for this apparent paradox could be linked to the destabilizing potential of the *BCL-2* mRNA adenine- and uracil-rich element, which can be regulated by different mechanisms [158]: half-life of the mRNA of *BCL-2* in Jurkat cells is prolonged by protein kinase C stimulation, but shortened by C(2)-ceramide addition. This supports the view that *BCL-2* mRNA stability plays a physiological role in modulating Bcl-2 levels. 

The expression of apoptosis-related genes (e.g., *BCL-2*, *P53*, *FAS*, NO synthetases, *etc.*) may affect tumor growth and possibly metastatic inefficiency [149]. Takaoka *et al.* observed that *bcl-2* overexpression in B16M cells enhanced pulmonary metastasis [147]. In parallel, melanoma cells resistant to fas-mediated apoptosis were found to be more susceptible to metastasize [148]. Furthermore, although apoptotic *H-ras* and *v-myc* transformed metastatic fibroblasts labeled with green fluorescent protein were observed in the lungs, *in vitro*-induced *bcl-2* overexpression in these cells conferred resistance to apoptosis 24–48 h after inoculation [150]. Thus, it is plausible that regulation of cell death mechanisms influences metastatic growth, at least in the early stages after attachment to the vascular endothelium. 

Further investigations showed that *BCL-2* was preferentially overexpressed in B16M-F10 cells as compared with the low metastatic F1 cell variant [142]. There was also a good correlation between increased gene expression and increased protein content, which, in these B16M cells, minimizes the effect of post-transcriptional regulation steps [142]. *In vitro* HSE-induced B16M-F10 cytotoxicity was almost 100% when GSH-depleted metastatic cells (BSO-treated; buthionine sulfoximine, a specific GSH synthesis inhibitor) were treated with a Bcl-2 antisense oligodeoxynucleotide (*bcl-2*-AS) [142]. Furthermore, when BSO- and *bcl-2*-AS-pretreated B16M-F10 cells were inoculated intravascularly into mice, the number of intact arrested cells on the HSE decreased by 98% and the very small number of metastatic cell survivors (probably bearing molecular damages) [142] were not able to develop detectable colonies [142]. This suggests that intravascular granulocytes and/or the Kupffer cells, present in the metastatic microenvironment, probably eliminate those few survivors. 

Recent reports show that, in addition to its anti-apoptotic properties, Bcl-2 can also inhibit multidrug resistance dependent-GSH efflux from metastatic cells [105,159], thus improving metastatic cell resistance against ROS, RNS, cytotoxic drugs and ionizing radiation. Consequently, based on *in vitro* and *in vivo* findings in B16M cells (on the interaction between endothelial and metastatic cells, and the effect of ROS and RNS), GSH and Bcl-2 appear candidates to challenge survival of cancer cells with high metastatic potential. Nevertheless, this approach must be validated for other tumor types, where other molecules may be additional critical targets.

## 4. Invasion and Colonization

### 4.1. Angiogenesis and Metastatic Growth

The development of new vessels from pre-existing ones is termed angiogenesis or neovascularization, and includes hemangiogenesis and lymphangiogenesis [160]. The induction of angiogenesis is a crucial early stage in the development and growth of most solid tumours, and is also necessary for haematogenous and/or lymphagenous dissemination of cancer cells [2,161].

Regarding metastases, after colonization, persistent growth of metastatic tumors requires the establishment of new vasculature to ensure nutrients and oxygen supply [58].

Despite studies in animal models indicating that tumor lymphangiogenesis was associated with metastasis, metastatic spread to lymph nodes in different models occurred in the absence of tumor lymphangiogenesis, presumably via pre-existing lymphatic vessels. This suggests that the importance of lymphangiogenesis in metastasis may vary, depending on parameters such as the tumor type, or the position of the primary tumor relative to the lymphatic network [162].

All blood vessels are lined with endothelial cells that must proliferate and migrate to tumoral cell targets [163]. Angiogenesis is also a complex multistep process of growth and remodeling involving degradation of the extracellular matrix, cell migration and proliferation, and tube formation [162].

Folkman showed that tumors cannot grow beyond 1-2 mm without new blood vessel formation [163], and suggested that dormant metastases fail to grow because of the lack of neoangiogenesis (“angiogenic dormancy”) [164]. Dormant metastases-associated senescent cells exhibited growth arrest (mainly in G1), likely due to the NO present in the microenvironment [8].

Angiogenesis is favored by a pro-angiogenic tumor microenvironment comprised of hypoxia, increased growth promoting factors/cytokines, decreased antiangiogenic factors, secretion of metalloproteinases (MMPs), and increased cycloxygenase-2 (COX-2) [163]

Although a vast variety of growth factors and cytokines act as inducers of angiogenesis, e.g., placental growth factor (PIGF), fibroblast growth factors (FGF-1 and -2), platelet-derived growth factor (PDGF), hepatocyte growth factor (HGF), angiopoietins (Ang-1 and -2), epidermal growth factor/transforming growth factor-α (EGF/TGF-α) [162]; the vascular endothelial growth factor (VEGF), due to its selectivity for endothelial cells, is the most specific growth factor for the endothelium [163]. VEGF induction in different tissue led to massive cell infiltration, predominantly hematopoietic cells, to tumor perivascular sites [165]. VEGF-A is a potent angiogenic factor with some (weak) prolymphangiogenic effect, whereas VEGF-C and VEGF-D are potent lymphangiogenic factors [166].

New vessels are highly irregular, weak, and have incomplete endothelial linings and membrane basement. Thus, adhesion of cancer cells to a new endothelium increases resistance to blood flow, may decrease tumor perfusion, and cause tumor hypoxia [160]. Under hypoxic conditions, e.g., in the central area of the tumor mass where oxygen pressure is low, tumor cells adopt anaerobic bacteria-like characteristics, including low levels of mitochondrial oxidative phosphorylation [167]. Increased glycolysis and diminished mitochondrial activity are universal mechanisms common to all tumor cell types with low O_2_ availability and generically known as the “Warburg effect” [167,168].

Lack of oxygen and nutrients, extracellular acidity (induced by the release of CO_2_ and lactic acid), and the up-regulation of glycolysis are all metabolic conditions that favor angiogenesis, invasion, metastasis, and mutation in *P53* [160].

Hypoxia in solid primary tumor tissues or the metastatic microenvironment, promotes stabilization and activation of hypoxia-inducible factor-1a (HIF-1a), which participates in activating erythropoiesis, glycolysis, angiogenesis, cell growth, metastasis, and p53, as well as in regulating transcription of ABC transporter genes and Bcl-2 [160]. Thus, establishing molecular links between hypoxia and tumor chemoresistance.

The proliferating tumor cells, their surrounding host stromal cells, and tumor-infiltrating inflammatory/immune cells create a tumor microenvironment that reflects a persistent inflammatory state [169]. This chronic inflammation is closely associated with angiogenesis [169].

During tumor growth or under circumstances of prolonged stress, such as chronic inflammation or hypoxia, cell heterotypic interaction plays an important role in driving tumor promotion. Among these cells are macrophages, neutrophils and lymphocytes [58,170]. 

Inflammatory cells secrete a large number of cytokines and chemokines. Cytokines, such as interleukin-1 (IL-1) and tumor necrosis factor (TNF), can act directly on endothelial cells favoring tumor cell adhesion. This effect is mediated, at least in part, through the synthesis and expression of specific endothelial cell surface binding proteins [171], and through production of autocrine growth factors by the tumor cells [172]. These proinflammatory cytokines activate ROS and RNS generation [172]. For example, TNF-α enhances the formation of ROS by neutrophils and other cells, while IL-1-β, TNF-α and IFN-γ stimulate the expression of iNOS in inflammatory and endothelial cells [172]. 

The macrophages infiltrating the tumor have a crucial role in regulating its progression [58]. The fibroblasts produce cytokines and proteases that can promote endothelial cell proliferation and might activate dormant cells and trigger angiogenesis and metastasis [58,173]. Macrophages, platelets, fibroblasts and tumor cells themselves are major sources of angiogenic factors such as bFGF, VEGF, prostaglandins-1 and -2, in addition to inflammatory cytokines, chemokines, and NO [172].

Excessive formation of NO in the cancer microenvironment or at inflammatory sites promotes enhanced vascular permeability, immune cell infiltration, and cytotoxicity [167]. In addition, NO reacts with O_2_^-^, generating ONOO^−^, which, besides damaging macromolecules, plays roles stimulating production of prostaglandins and activating MMPs. Prostaglandins induce the expression of several pro-inflammatory cytokines, which further enhance the production of ROS and RNS [167].

Free radicals react with membrane phospholipids generating hydroperoxides, lipoperoxides, and toxic aldehydes such as MDA, which in turn induce further adhesion of granulocytes to the endothelium and activation of intracellular pathways that produce more free radicals [172]. This molecular cascade causes DNA damage, post-translational modification of key oncoproteins, suppression of DNA repair enzymes, promotion of cell proliferation, inhibition of apoptosis, angiogenesis and metastasis, and suppression of host antitumor defense [169].

Lipoperoxides, such as the 4-HNE, form adducts with DNA, e.g., at codon 249 of the *P53* gene [169], and at high concentrations induce DNA oxidation and apoptosis, and upregulate *COX-2* expression [172]. COX-2 is a main enzyme induced by prostaglandins in inflammatory cells such as monocytes and macrophages (in cytokine-stimulated macrophages, iNOS enhanced the activity of COX-2 *via* S-nitrosylation); and non-inflammatory cells such as fibroblasts and endothelial cells [172]. COX-2 also plays a role in cancer development through angiogenesis (increased expression of VEGF, promotion of vascular sprouting, migration and tube formation, induction of MMPs, and activation of EGFR) [169]; and through the activation of different oncogenes, including *SRC*, *RAS*, *HER-2* and *WNT* [172]. 

The peroxidation of cell membranes is regulated by lipoxygenases (LOXs) and the phospholipid hydroperoxide-glutathione peroxidase or glutathione:lipid-hydroperoxide oxidoreductase (PH-GPx). LOXs catalyze the specific dioxygenation of polyenoic fatty acids, forming reactive fatty acid hydroperoxides. PH-GPx reduces organic and inorganic hydroperoxides by utilizing GSH as a reducing agent. LOXs have been found in different cancer tissues, including melanoma, prostate, and epidermal cancers, and their expression also correlates with tumor cell metastatic activity [172]. 

### 4.2. Dynamic Adaptations and the Road to Perdition

Studies on the organ distribution of B16M cells showed that less than 1% of all circulating cancer cells survive and may promote metastases [174]. Indeed, the majority of cancer cells entering the microvascular bed of the liver [175] and other organs [94] are killed within the first hours due to blood flow-associated mechanical trauma [176], their inability to withstand deformation [175], locally released ROS/RNS [133], and their susceptibility to the lytic action of immunocompetent intrasinusoidal lymphocytes and macrophages [177]. Thus, cancer spread and invasion of secondary tissues/organs appears poorly effective, a fact biologically expressed as ‘‘metastatic inefficiency” [103,178,179], which implies that only highly resistant cell subsets begin metastatic invasion and start secondary growth. Whether these cells correspond, totally or partially, to the so-called cancer stem cells (CSCs) is an open question. The marked resistance of CSCs towards classical antitumor regimens is mediated by the combination of several critical features, including relative dormancy, efficient DNA repair, high expression of multidrug-resistance-type membrane transporters and protection by a hypoxic niche environment [180]. Therefore, it is possible that invasive metastatic cells may represent a (heterogeneous?) subset highly enriched in CSCs.

Affecting invasive cells, high NO and H_2_O_2_ levels are damaging and eventually may cause cell death [44,131]. Nevertheless ROS and RNS often function as a double-edged sword, causing cell death when in excess (*i.e.,* during interaction of cancer and endothelial cells) or protecting cells against apoptotic or necrotic inducers when present at physiological levels (*i.e.*, during secondary metastatic growth within an invaded tissue). Indeed, stress can induce genetic and epigenetic alterations leading to the expansion of new tumor cell populations [181]. 

On the molecular background of this mechanism, microarray-based gene expression analysis revealed changes in approximately 2000 transcripts in the response of e.g., HL60 cells to low H_2_O_2_ concentrations in particular, including 200 GSH-related genes [182]. Some of these overexpressed genes are key factors in carcinogenesis, such as NF-κB activation or DNA methylation, genes for cytokine and chemokine ligands and receptors, the redox regulator thioredoxin interacting protein, the histone deacetylase sirtuin, heat-shock proteins (e.g., HSP40 and HSP70) and the AP-1 transcription factor components Fos and FosB [182]. Moreover, ROS affect *HIF-1* and *VEGF* expression in cancer cells (e.g., [183]), and are involved in regulating angiogenesis and tumor growth [181,184]. On the other hand, NO also acts on several signaling pathways directly activating transcription factors, such as NF-κB or AP-1, and thereby influences gene expression [43]. Nevertheless, RNS, in some cases, indirectly modulates activity or stability of e.g., HIF-1 or NF-κB, or modulates accessibility of promoters via increased DNA methylation or histone deacetylation [185].

In B16M-F10 cells interacting with endothelial cells, H_2_O_2_- and NO-induced adaptations include cell adhesion molecule expression in both endothelial and cancer cells (see above), activation of the early growth response-1 transcription factor gene, activation of cancer and endothelial cell MMPs, increase of antioxidant enzymes such as MnSOD and catalase, and induction of key invasive growth-related molecules such as VEGF-A, HIF-1 and protein 8 [159,186]. Moreover, although high NO levels down-regulate the antiapoptotic protein Bcl-2 and induce cytotoxicity in e.g., B16M-F10 cells [187], low NO levels induce S-nitrosylation of Bcl-2, which inhibits its ubiquitination and subsequent proteosomal degradation in, for example, lung cancer cells. Facts, among others, implicating NO as a potential key regulator of cell death resistance [188,189]. 

Therefore, although many molecular factors are involved, ROS and RNS are among those favoring selection and growth of highly resistant metastatic cells, and thus paving the way to patient perdition. ROS and RNS generation is not constant and their levels are variable under *in vivo* conditions, thus this process of selection must be dynamic. This implies that changing conditions within the tissue microenvironment, systemic/intraorgan signals, immune cells attacks, or therapy-related cancer cell stress, may cause a constant change in the genomic/proteomic profile of metastatic growing survivors. Therefore, efforts focusing studies on the biology of these highly resistant cell subsets could be key to improving the efficacy of cancer therapy.

## 5. Therapeutic Implications

The effective treatment of highly resistant tumor cells, frequently small metastatic subpopulations, is consequently a priority of anticancer therapy. This notion is important due to the different efficacy rates observed in patients with apparently the “same tumor” (type, stage) and receiving the same treatment. Therefore, to improve cancer treatment efficacy it appears also necessary to better work out (a) early diagnosis; (b) prediction of tumor evolution and response to treatment; (c) drug/radiation dosage and time course, and (d) toxicity-derived side effects. Gene expression and pathway signatures might help in these tasks. This includes the development of some RT-PCR and microarray based multigene tests, *i.e.*, which in breast cancer include: MammaPrint, Oncotype DX, BLN Assay, Theros Breast Cancer Index SM, MapQuant DX, ARUP Breast Bioclassifier, Celera Metastatic Score, eXagen BCtm, Invasive Gene Signature, Wound Response Indicator and Mammostrat. Two of these (Oncotype DX and MammaPrint) have been already incorporated into several diagnostic protocols. Nevertheless, the high diversity of ROS- and RNS-induced effects, and the influence of the complex tumor microenvironment (including spatial and temporal constraints) [59,67], makes it difficult to establish a possible oxidative-nitrosative signaling signature [190].

Low levels of NO have been associated with increased cGMP-mediated ERK phosphorylation, a response that favors a pro-growth and anti-apoptotic behavior. However, these prosurvival effects are lost at higher NO concentrations, which increase phosphorylation and acetylation of p53, while low levels stimulate proliferation [191,192] and promote invasion and metastasis [193,194,195,196]. 

The dual role of ROS and RNS in oncology- both anti-neoplastic and pro-neoplastic - clearly suggest that the type of tumor, its cellular redox state, as well as the final concentration and the duration of exposure to ROS and NO, may be determinant. Based on these facts, a number of therapies, drugs and approaches have been developed. On one hand, antioxidant and antinitrosative compounds may contribute to protect the organisms against cancer initiation. In this sense, for instance, a lot of natural compounds with antioxidant activity have potential applications, e.g., curcumin [197,198], resveratrol [199,200,201,202], pterostilbene [203,204], epigallocatechin-3-gallate [205,206], or quercetin [207,208]. In addition, although toxic effects were found, the use of the NOS inhibitor N-nitro-L-arginine in a single dose reduced the tumor blood volume in patients [209].

On the other hand, oxidative and nitrosative stress can contribute to tumor removal. In fact, many chemotherapeutic agents, as well as ionizing radiation, increase ROS and RNS production up to cytotoxic levels [59,210,211]. Different NO donors have been also proposed [120,212]. However, natural compounds (as those mentioned above) may increase cancer sensitivity to e.g., chemotherapy or induce cancer demise [187,213,214,215,216,217,218,219].

Moreover, ionizing radiation has been shown to increase drug delivery and therapeutic efficacy through an NO (increase)-dependent transient tumor reoxygenation [120].

Hence, NO properties as vasodilator, inhibitor of cell respiration, radiosensitizer, modulator of tumor immunity, and angiogenesis stimulator can be utilized to improve the efficacy of conventional antitumoral treatments. Nevertheless, our partial understanding of how RNS and ROS act in the tumor microenvironment implies limitations in order to develop effective treatments. Further basic research, as well as new NO donors, NOS agonists, modulators of tumor antioxidant defenses, patient selection, and combined treatments, should help to improve the efficacy of cancer therapy.

## 6. Future Directions

ROS and RNS contribute to maintaining the malignant phenotype. Thus, further research is necessary to improve our understanding of the complex mechanisms that regulate their roles in tumor biology. Cancer cells exposed to low levels of these reactive species, or capable of resisting ROS/RNS-mediated injury, undergo a clonal selection that favors their survival. In this mechanism, activated oncogenes and/or inactivated tumor suppressor genes may result in activation of multiple transcription factors. At advanced stages, uncontrolled tumor growth and the development of stress conditions, such as hypoxia, acidosis, inflammation, and free radical overproduction, may further alter the activity of these transcription factors. These events may cause aberrant expression of multiple metastasis-related proteins and confer survival and growth advantages to metastatic cells. However, it is essential to take into account that metastatic cells are surrounded by the tumor stroma or microenvironment, which includes resident non-cancerous cells (fibroblasts, glial cells, epithelial cells, adipocytes, inflammatory cells, immunocytes, and vascular cells), connective tissue, extracellular matrix, and extracellular molecules. At present, there is a limited understanding of the complex relationship between metastatic cells and the surrounding host cells. For years researchers have focused on the cancer cell itself, but it is now acknowledged that metastatic cells and their stroma co-evolve during tumor progression. Nevertheless, how normal cells or newly recruited cells are altered during metastatic progression, and how they reciprocally influence metastatic growth are poorly understood. Therefore, our knowledge on these questions must improve to permit the development of therapeutic strategies targeted at both the microenvironment and the tumor. Moreover, it may be possible to develop strategies to prevent metastatic growth based on our understanding of how alterations in the microenvironment affect that growth. Available technologies will provide the tools for a better understanding of the tumor microenvironment and for the development of tissue- or cell-specific targeting agents. In this sense, isolation and characterization of both metastatic and stromal cells appears critical in order to learn how to make the tumor microenvironment hostile to the tumor.

It is in this scenario where a complex balance between pro- and anti-metastatic ROS and RNS (possibly involving other cancer-, endothelium-or immune cell-derived cytotoxic/signaling molecules) regulate the progression of metastatic cells. Thus, gene expression, cell signaling, and proteomic profiles may help to identify (a) key targets involved in metastatic cell escape mechanisms, and (b) RNS- and ROS-induced adaptive responses in selected pools of invasive cells, in particular those that resist aggressive anti-cancer treatments.
